# Extensive Cerebral Venous Sinus Thrombosis With Obstructive Hydrocephalus in a Young Female With Underlying Essential Thrombocythemia: A Case Report

**DOI:** 10.1002/ccr3.73669

**Published:** 2026-09-30

**Authors:** Sarah Nafea, Tasmia Farooq, Md Abu Taher, Andrea Barros, Kyaw Naing Soe, Fawad Ali, Koshy Thomas

**Affiliations:** ^1^ Department of Acute Medicine Basildon and Thurrock University Hospital, Mid and South Essex NHS Foundation Trust Essex UK; ^2^ Department of General Medicine Jalalabad Ragib Rabeya Medical College Sylhet Bangladesh

**Keywords:** case report, cerebral venous sinus thrombosis, essential thrombocythemia, JAK2 V617F, obstructive hydrocephalus

## Abstract

Persistent symptoms suggestive of idiopathic intracranial hypertension despite standard therapy should prompt urgent neuroimaging to exclude cerebral venous sinus thrombosis. When CVST is confirmed without an obvious provoking factor, evaluation for underlying myeloproliferative neoplasms, including JAK2‐positive essential thrombocythemia, is essential.

## 1 Introduction

Cerebral venous sinus thrombosis is a rare form of stroke that predominantly affects younger people and accounts for around 0.5%–3% of all cerebrovascular events [1]. Recent epidemiological reports indicate that CVST has risen over the last 10 years, mainly due to increased clinical awareness and greater access to advanced neuroimaging techniques such as magnetic resonance venography [2, 3]. Despite these developments, the diagnosis remains challenging because of the common occurrence of isolated headache and the delay in diagnosing and recognizing cerebral venous thrombosis [4, 5]. Early diagnosis enables early treatment of CVST with anticoagulation, the most important treatment, and is associated with good functional outcomes in most patients [6].

Its clinical manifestation is very variable, from a sudden, continuous headache (often the only symptom for days or weeks) to a focal neurological deficit, seizures, and altered consciousness. Identifying CVST in early stages is often difficult; the most common misdiagnosis being idiopathic intracranial hypertension (IIH), which is caused by raised ICP, visual disturbance, and papilloedema [7].

Physiopathologically, thrombotic obstruction of the cortical veins and cerebral venous sinuses impairs the normal venous drainage, resulting in local edema, venous hypertension, and severe hemorrhagic venous infarction. Deep cerebral venous system involvement is far less frequent than superficial system thrombosis. Still, it tends to follow a more fulminant course, as these vessels drain the periventricular white matter, hypothalamus, thalami, and basal ganglia [8].

Myeloproliferative neoplasms (MPNs) are acquired prothrombotic disorders that commonly lead to CVST. Essential thrombocythemia is a form of MPN characterized by increased production of abnormal platelets, further amplified by the JAK2 V617F mutation in 50%–60% of patients. The combination of this excessive, abnormal platelet count with disordered leukocyte and erythrocyte pathology creates a highly prothrombotic state at unusual arterial or venous circulatory sites [1]. Venous thromboembolism is a well‐known complication of essential thrombocythemia; however, cerebral venous sinus thrombosis is a rare complication [9, 10]. The presence of the JAK2 V617F mutation is associated with an increased risk of thrombosis and is an important determinant of thrombotic risk stratification in patients with essential thrombocythemia [11]. Thus, the current guidelines recommend evaluating for underlying myeloproliferative neoplasms (MPNs), including testing for JAK2 mutations, in patients with CVST who have no obvious provoking factors [12].

Obstructive hydrocephalus as a direct complication of CVST is a rarely reported clinical finding [8]. Hydrocephalus in the setting of CVST may result from impaired CSF resorption related to elevated venous pressure and, in some cases, from ventricular obstruction associated with venous congestion and cerebral edema [13]. Early diagnosis is of great clinical significance, as it can lead to the worsening of intracranial hypertension and may require urgent neurosurgical intervention.

We present a rare case of extensive cerebral venous sinus thrombosis with both superficial and deep venous involvement in a young woman with previously undiagnosed JAK2 V617F‐positive essential thrombocythemia, who developed bilateral thalamic infarction and obstructive hydrocephalus. The initial presentation closely resembled idiopathic intracranial hypertension, resulting in an initial diagnostic impression of IIH before subsequent neuroimaging established CVST. Therefore, in such patients, a high index of suspicion for CVST is warranted, and prompt neuroimaging, detailed etiological workup, and a multidisciplinary approach to management are crucial.

## 2 Case History/Examination

A 30‐year‐old woman without any past medical or surgical history presented with worsening symptoms of persistent headache, severe vomiting, and visual disturbance. At initial presentation to a secondary‐level clinic, a primary diagnosis of IIH was made due to increased CSF opening pressure revealed on lumbar puncture. Treatment was started with acetazolamide, with a little symptomatic improvement initially.

However, over a few days and weeks, her condition started to get worse with increased intensity of headache, projectile vomiting, and progressive visual blurring in both eyes. Upon arrival at a specialized healthcare facility, urgent neuroimaging was planned. The brain CT report showed mild dilatation of the lateral ventricles, prominence of the temporal horns, and a hyperdensity in the area of the deep venous structures, consistent with hydrocephalus and venous sinus thrombosis (Figure 1a,b).

**FIGURE 1:**
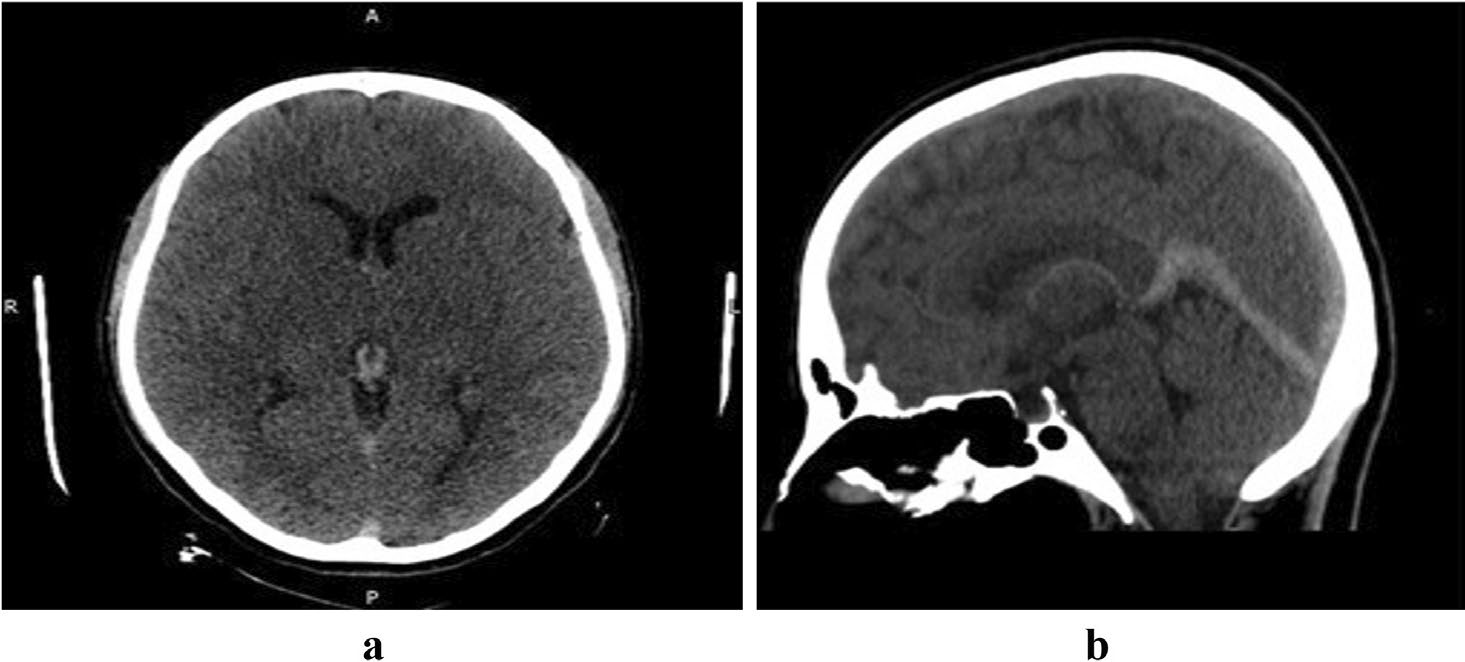
Noncontrast CT (a) Axial section showing mild ventricular prominence and venous sinus thrombosis. (b) Sagittal section showing a linear hyperdensity along the superior sagittal sinus (hyperdense cord sign) consistent with acute CVST.

A magnetic resonance imaging (MRI) scan was also done. Axial T2‐weighted imaging showed thalamic hyperintensity bilaterally with enlargement of lateral ventricles. FLAIR imaging further showed abnormalities in the bilateral thalamic signal with periventricular changes. Diffusion‐weighted imaging also showed restricted diffusion in both thalami due to deep venous thrombosis. Bilateral temporal horn dilation was also noted, all of which together further supported the findings of extensive thrombosis complicated by obstructive hydrocephalus (Figure 2a–d).

**FIGURE 2:**
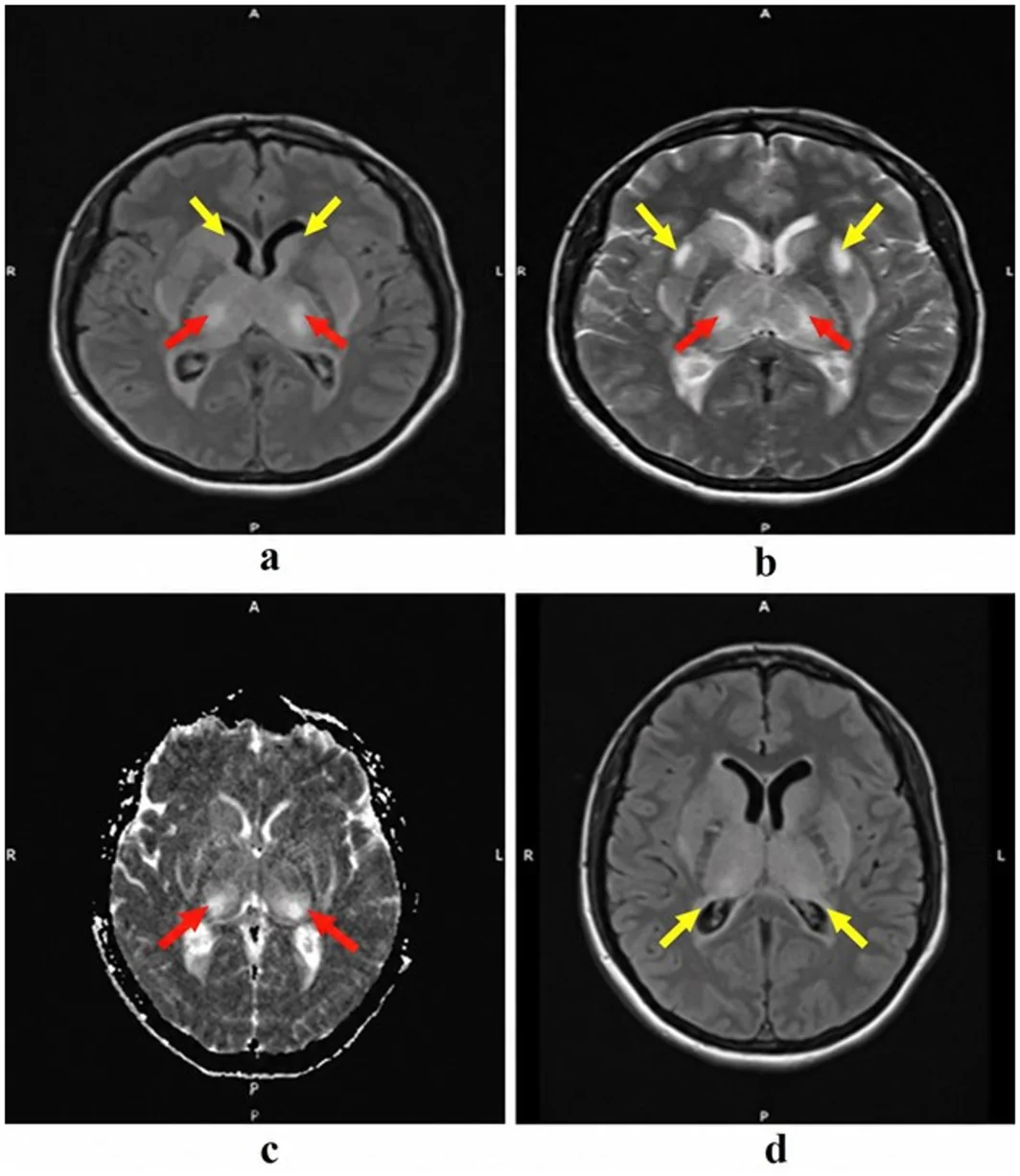
MRI brain. (a) T2: Bilateral thalamic hyperintensity with mildly prominent ventricular appearance. (b) FLAIR: Confluent bilateral thalamic signal change with periventricular edema. (c) DWI: Bilateral thalamic restricted diffusion consistent with venous infarction. (d) Axial T2: Bilateral temporal horn dilation confirming hydrocephalus.

On further magnetic resonance venography (MRV) imaging scans, the coronal MIP views showed no flow in the superior sagittal sinus to very little flow in the transverse sinuses. Sagittal MIP images confirmed extensive thrombosis of both superficial and deep systems. Prominent cortical collaterals were also noted, suggesting a compensatory rerouting of venous flow (Figure 3a,b).

**FIGURE 3:**
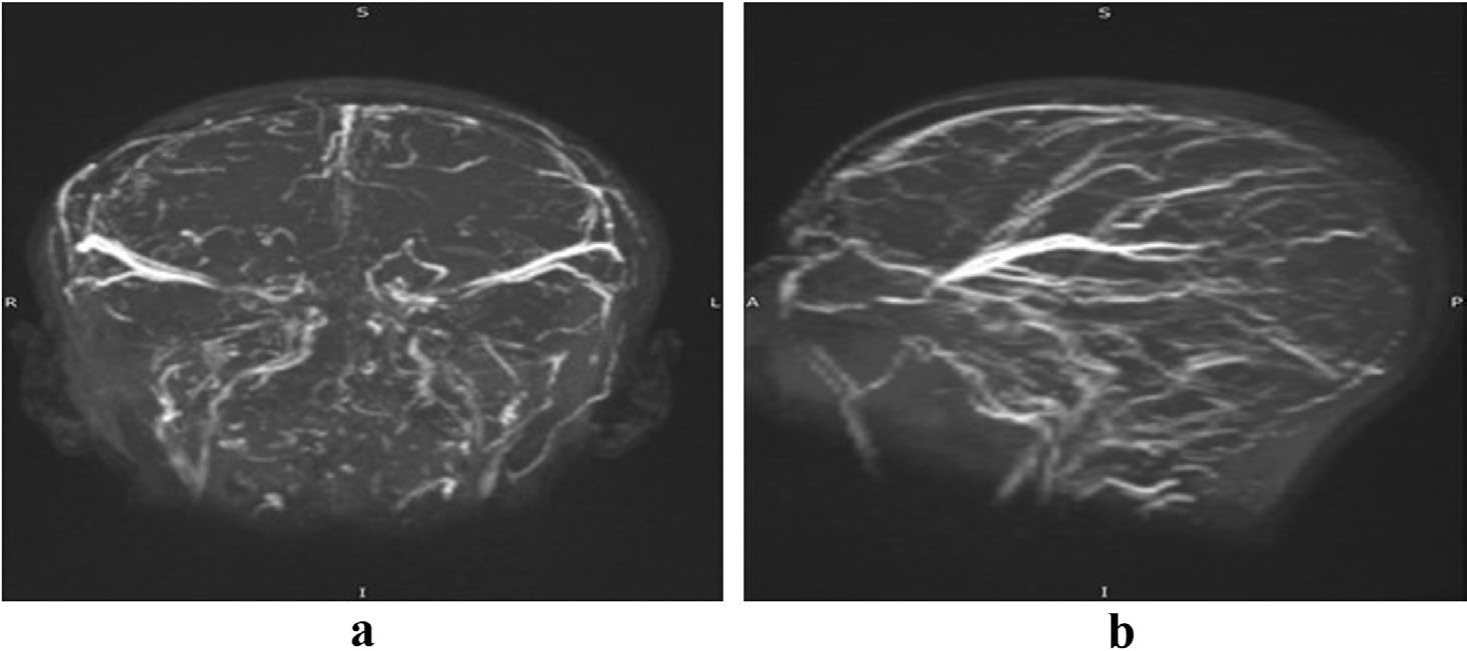
Pretreatment magnetic resonance venography (MRV). (a) Coronal MIP image demonstrating extensive thrombosis with markedly reduced flow in the superior sagittal and bilateral transverse venous sinuses. (b) Sagittal MIP image confirming extensive thrombosis of the superficial and deep cerebral venous systems with prominent collateral venous drainage.

## 3 Methods

### 3.1 Differential Diagnosis

Persistent headache, visual disturbance, and elevated CSF opening pressure were the reasons for the initial diagnosis of idiopathic intracranial hypertension. However, this progressive deterioration on treatment with acetazolamide led to further neuroimaging, which confirmed the diagnosis of cerebral venous sinus thrombosis.

### 3.2 Investigations

Complete blood count showed persistent marked thrombocytosis, with a platelet count of 1140 × 10^9^/L [9] on repeated measurements. Peripheral blood smear demonstrated large platelets. Bone marrow biopsy demonstrated hypercellularity with megakaryocytic hyperplasia. The available pathology report did not provide further morphological details. Molecular testing identified the JAK2 V617F mutation with a variant allele frequency of 14%. CALR and MPL variants were not detected, while BCR::ABL1 transcripts were not detected. Available investigations did not identify a secondary cause of thrombocytosis.

### 3.3 Treatment

Immediate anticoagulation was initiated with low‐molecular‐weight heparin. A multidisciplinary assessment plan was developed, including the departments of ophthalmology, neurology, radiology, and hematology. Ocular assessment showed bilateral papilledema. Continuous intracranial pressure monitoring was started. The hematology team was involved to identify the underlying cause of the thrombotic event.

Immediate anticoagulation was initiated with enoxaparin 80 mg on 21 April 2026 and was continued until transition to apixaban in May 2026. No separate apixaban loading dose was documented; apixaban was initiated at 5 mg twice daily in May 2026 and was continued thereafter. Given the extensive CVST and underlying ET, long‐term anticoagulation was considered appropriate.

## 4 Conclusions and Results

### 4.1 Outcome

The patient showed progressive clinical improvement after starting anticoagulation and cytoreductive therapy, with normalization of the platelet count and no new thrombotic events during follow‐up.

### 4.2 Follow‐Up

On follow‐up on 2 June 2026, the patient showed clinical improvement, and the platelet count had decreased to 355 × 10^9^/L [9] from 1140 × 10^9^/L [9] at presentation. Hemoglobin was 124 g/L, hematocrit was 0.41 L/L, and white blood cell count was 5.6 × 10^9^/L [9]. Follow‐up magnetic resonance venography demonstrated partial recanalization of the right transverse sinus and the posterior aspect of the superior sagittal sinus, which remained incompletely recanalized (Figure 4a,b). Susceptibility‐weighted imaging showed blooming in the left basal ganglia, suggestive of blood products. No clinically significant bleeding complication was documented during anticoagulant therapy. Otherwise, the brain appeared unremarkable on the follow‐up examination. No new thrombosis was observed during subsequent follow‐up, and the patient remained under multidisciplinary neurological and hematological care. Table 1 summarizes the clinical course, diagnostic evaluation, treatment, and follow‐up.

**FIGURE 4:**
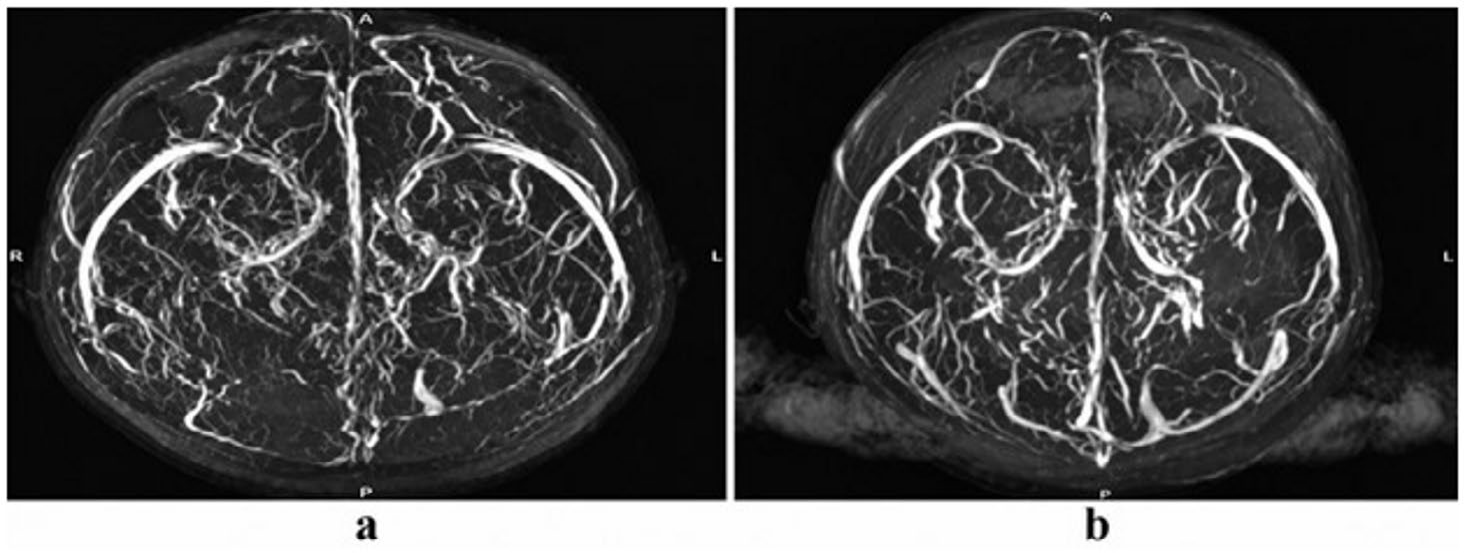
Follow‐up magnetic resonance venography (MRV). (a) Coronal maximum‐intensity projection (MIP) image demonstrating partial recanalization of the superior sagittal sinus, with restoration of venous flow following treatment. (b) Coronal MIP image showing improved opacification of the bilateral transverse sinuses compared with pretreatment imaging, consistent with interval radiological improvement.

**TABLE 1:** Clinical timeline of presentation, diagnostic evaluation, treatment, and follow‐up.

Date	Clinical events, investigations, and management
1 April 2026	Initial presentation with persistent headache, severe vomiting, and visual disturbance.
1 April 2026	Lumbar puncture demonstrated increased CSF opening pressure, and idiopathic intracranial hypertension was initially diagnosed.
1–7 April 2026	Acetazolamide was initiated, with limited initial symptomatic improvement.
8–20 April 2026	The patient developed worsening headache, projectile vomiting, and progressive bilateral visual blurring.
21 April 2026	CT, MRI, and MR venography demonstrated extensive CVST involving superficial and deep venous systems, bilateral thalamic abnormalities, and obstructive hydrocephalus.
21 April 2026	Low‐molecular‐weight heparin was initiated. Ophthalmological assessment demonstrated bilateral papilledema, and multidisciplinary evaluation was undertaken.
22–30 April 2026	Hematological evaluation demonstrated marked thrombocytosis (platelet count, 1140 × 10^9^/L). Bone marrow examination demonstrated megakaryocytic hyperplasia, and molecular testing identified JAK2 V617F positivity (VAF, 14%), supporting a diagnosis of essential thrombocythemia.
May 2026	Anticoagulation was transitioned to apixaban, and cytoreductive therapy with pegylated interferon alfa was initiated.
2 June 2026	Follow‐up laboratory evaluation demonstrated a platelet count of 355 × 10^9^/L, compared with 1140 × 10^9^/L at presentation. Hemoglobin was 124 g/L, hematocrit 0.41 L/L, and white blood cell count was 5.6 × 10^9^/L.
June 2026	Follow‐up MR venography demonstrated partial sinus recanalization, with restoration of flow in the superior sagittal sinus and improved opacification of the bilateral transverse sinuses.
Subsequent follow‐up	No new thrombosis was observed, and the patient remained under multidisciplinary neurological and hematological care.

## 5 Discussion

This case highlights several clinical and diagnostic problems commonly encountered in CVST but rarely seen in the same patient. Two related findings are of particular importance: overlap among IIH, deep venous thrombosis, and their associated life‐threatening complications; and the pathophysiology and advanced treatment of myeloproliferative neoplasms with JAK2‐positive features. New neuroimaging techniques and growing clinical awareness have improved the diagnosis of CVST, but the clinical features remain nonspecific and variable, requiring a high index of clinical suspicion [13]. There is recent research that identifies delayed diagnosis, particularly when the presenting symptoms are headaches alone or when the presenting symptoms are comparable to idiopathic intracranial hypertension [4, 14]. If the diagnosis is delayed, it may allow thrombus propagation, which can lead to venous infarction, hydrocephalus, and neurologic deterioration [15, 16].

The initial diagnosis of IIH at first presentation in this patient is of great clinical significance. The primary signs and symptoms of both disorders are similar and include vomiting (which is progressive), headache that gradually worsens, visual disturbance, and raised CSF pressure on lumbar puncture. The clinical course of IIH and CVST also differentiates these two conditions: acetazolamide will often help improve IIH, whereas CVST will worsen over time. Therefore, if a patient is labeled with a diagnosis of IIH and the symptoms get worse even after initial therapy with acetazolamide, then neuroimaging with MR venography should be planned urgently, because plain CT may be unable to detect venous sinus thrombosis in up to one third of cases [7]. Patients with suspected IIH should be screened with MRI and magnetic resonance venography for cerebral venous sinus thrombosis as part of the diagnostic process, per recent recommendations [17]. Our patient's progressive symptoms despite acetazolamide therapy represented an important clinical clue that prompted further imaging and ultimately established the correct diagnosis.

The widespread thrombosis was remarkable in this case, with anatomical involvement extending to the superior sagittal sinus, both transverse sinuses, both sigmoid sinuses, and the right internal jugular vein, reflecting complete involvement of the supratentorial venous drainage system. The grave finding of thrombosis in the deep venous system added to it. The internal cerebral veins and the vein of Galen both drain the basal ganglia, thalami, hypothalamus, and periventricular white matter. These areas are devoid of collateral flow and predisposed to infarction due to the rapid development of congestion [13]. The prognosis of CVST with hydrocephalus is poorer, and mortality rates are higher, but the link between the two remains uncertain [9]. In most cases, bilateral thalamic and basal ganglia lesions can result in high morbidity or death. In this striking case, the patient's neurological recovery is a testament to both the young brain's resilience and the importance of early diagnosis and tailored management.

Hydrocephalus in the presence of CVST warrants special attention, as it is infrequently reported in the current literature [8]. Two mechanisms may contribute to ventricular enlargement in this setting. First, increased venous sinus pressure can impair CSF resorption by altering the pressure gradient across the arachnoid granulations, potentially contributing to a communicating component. Second, venous congestion and edema involving the bilateral thalami and adjacent periventricular structures may contribute to ventricular obstruction [18]. In the present case, bilateral thalamic abnormalities, ventricular enlargement, and prominent temporal horns were demonstrated on CT and MRI. However, the available imaging does not allow definitive localization of the anatomical site of obstruction to the foramina of Monro or the cerebral aqueduct. Therefore, the hydrocephalus was considered likely multifactorial rather than attributable to a definitively demonstrated single obstructive site. Careful neuroimaging assessment is important in patients with CVST and suspected hydrocephalus to characterize ventricular changes and assess for progression [8]. An etiology‐based approach to hydrocephalus emphasizes careful neuroimaging assessment to identify the underlying anatomical cause and potential site of obstruction [19].

An important feature of the present case was the management of obstructive hydrocephalus without documented neurosurgical CSF diversion or decompressive intervention. The patient improved following treatment directed at the underlying venous thrombosis and associated hematological disorder. This observation emphasizes that management should be individualized according to neurological status, imaging progression, and response to medical therapy, while recognizing that patients with worsening intracranial pressure or neurological deterioration may require urgent neurosurgical intervention.

The finding of underlying JAK2‐positive ET provided an important underlying prothrombotic condition in this patient and influenced the long‐term management strategy. Essential thrombocythemia is an important prothrombotic condition associated with venous thrombosis, including thrombosis at unusual sites such as the cerebral venous circulation. The JAK2 mutation activates the JAK–STAT pathway, increasing platelet production and producing functionally abnormal platelets, both of which contribute to hypercoagulability [1]. The JAK2 V617F mutation contributes to endothelial dysfunction, leukocyte activation, and chronic inflammation, which significantly increase the risk of thrombosis in addition to thrombocytosis [20, 21]. Current evidence supports evaluation for underlying myeloproliferative neoplasms, including JAK2 V617F mutation testing, in patients with CVST without an obvious provoking factor [22]. CVST in relation to JAK2‐positive myeloid neoplasms has been reported in combination with other MPNs, such as prefibrotic primary myelofibrosis [23]. To our knowledge, the combination of extensive CVST, obstructive hydrocephalus, and JAK2 V617F‐positive essential thrombocythemia is rarely reported.

Given the persistent prothrombotic risk associated with essential thrombocythemia and the extensive nature of the CVST, long‐term anticoagulation was considered appropriate in this patient. Anticoagulation duration should be individualized according to thrombotic risk, bleeding risk, and hematological disease control [9, 24]. Management with pegylated interferon alpha for ET is highly significant for long‐term patient outcomes. It is preferred over other cytoreductive therapies in women of childbearing age because of its disease‐modifying potential reported in the current literature [9, 19].

This case report has inherent limitations because it describes a single patient, limiting the generalizability of the findings. The temporal relationship and biological plausibility of the development of extensive CVST and obstructive hydrocephalus from JAK2 V617F‐positive essential thrombocythemia are strong, but cannot be proven to be causal. More research is required to clarify this rarely reported clinical syndrome.

This case underscores the need to promptly obtain neuroimaging and expediently begin anticoagulation therapy in patients with elevated ICP who do not respond to initial treatment for IIH. A detailed workup should be done to identify underlying prothrombotic conditions, including essential thrombocythemia and other myeloproliferative neoplasms, if CVST is confirmed. Consultation with the neurology, hematology, radiology, and ophthalmology teams is essential to achieve the best results for patients. This report adds to the sparse literature on ET with JAK2 V617F by presenting an extensive CVST complicated by obstructive hydrocephalus and underscores the need for a high index of suspicion for rare causes of CVST.

## Author Contributions

**Sarah Nafea:** conceptualization, investigation, data curation, writing – original draft, writing – review and editing. **Tasmia Farooq:** conceptualization, investigation, data curation, writing – original draft, writing – review and editing. **Md Abu Taher:** conceptualization, supervision, writing – review and editing. **Andrea Barros:** investigation, validation, writing – review and editing. **Kyaw Naing Soe:** investigation, validation, writing – review and editing. **Fawad Ali:** investigation, validation, writing – review and editing. **Koshy Thomas:** supervision, validation, writing – review and editing.

## Funding

The authors have nothing to report.

## Ethics Statement

Ethical approval was not required for this single‐case report under institutional policy.

## Consent

We obtained written informed consent from the patient for publication of this case report and the accompanying images.

## Conflicts of Interest

The authors declare no conflicts of interest.

## Data Availability

Data sharing not applicable to this article as no datasets were generated or analysed during the current study.
